# Strategies and Best Practice in Cloning Small RNAs

**Published:** 2020-08-03

**Authors:** Hui Dai, Weifeng Gu

**Affiliations:** Department of Molecular, Cell and Systems Biology, UC Riverside 900 University Ave, Riverside CA 92521, USA

**Keywords:** Small RNA, High-throughput sequencing, Gene expression, RNA library

## Abstract

High-throughput sequencing has become a standard and powerful tool for analyzing nucleic acids primarily due to its sensitivity and convenience. Small RNAs play important roles in regulating cellular and viral genes. The conventional methods for small RNA analyses are tedious and often lack accuracy, specificity and sensitivity for many small RNA species. Therefore, high-throughput sequencing becomes an indispensable tool for analyzing small RNAs. However, it is challenging to generate a reliable and representative small RNA library for high-throughput sequencing since small RNAs are usually expressed at extremely low levels and often contain modifications which affect library construction, usually causing biased readouts. This review compares various strategies for generating small RNA libraries of high quality and reliability, and provides recommendations on best practice in preparing high-throughput sequencing RNA libraries.

## INTRODUCTION

The discovery of the first miRNA *lin-4* about 30 years ago by Victor Ambros lab may seem unimportant and irrelevant to human health since it is not conserved [1]. However, it inevitably opened a new era in small RNA-mediated gene regulation, antivirus and gene-editing [1–15]. It once again proves that any biological techniques humans developed may have been utilized by cells for millions of years. In this case, scientists used the base-pairing rule to develop hybridization methods for detecting nucleic acids before realizing that cells use the same strategy to screen target nuclei acids. Apparently cells outsmart humans since cells need to balance between detection sensitivity and specificity by using small RNAs while humans are more concerned with sensitivity by using long RNA probes. Unlike conventional small RNA species including tRNAs, 5S and 5.8S rRNAs, snRNAs, and snoRNAs, the newly discovered small RNAs are much smaller, only bearing 20–30 nucleotides (nt), and much less abundant.

Among them, micro(mi)RNAs and small interfering RNAs (siRNA) are processed from double-stranded (ds)RNAs in RNA interference (RNAi)-related processes, which regulate host and viral genes transcriptionally and post transcriptionally [1–3,16,17]; Piwi-interacting RNAs (piRNA) are processed from single-stranded (ss)RNA precursors and play critical roles in maintaining genome stability in germline cells [18–22]; promoter-associated small RNAs (PASR) or capped small RNAs (csRNA) are generated during Pol II-mediated transcription initiation, thus bearing a 5’ cap structure, and play important roles in regulating cellular and viral genes [23–26]. In addition, small RNAs can be generated by RNA degradation in cells and/or during RNA isolation/storage.

Unlike intact or fragmented mRNAs, small RNAs often contain distinct modifications usually at their 5’ and 3’ ends, which may block RNA ligation for constructing small RNA libraries. For example, animal miRNAs usually contain a monophosphate (p) group at the 5’ end (5’p-RNA) and 3’ hydroxyl (OH) group at the 3’ end resulted from Dicer-mediated processing [1,2,17]; plant miRNA and siRNAs are mostly 2’-O-methylated at the 3’ end for protection [27]; animal piRNAs are also 2’-O-methylated at the 3’ end for protection [19,28]; C. elegans endogenous 22G-RNAs contain a 5’ triphosphate (ppp) group (5’ppp-RNA) since they are made by RNA-dependent RNA Polymerases (RdRP) [29,30]; csRNAs contain a 5’ cap structure (Gppp) since they are made by cellular RNA Pol II [23,25,26]; degraded RNAs often contain a 5’ OH group at the 5’ end and a 2’−3’ cyclic phosphate or 2’ phosphate or 3’ phosphate at the 3’ end since most RNases generate products with such end features. The 2’-O-methylation can partially inhibit RNA ligation, reverse transcription (RT), and poly (A) polymerase (PAP)-mediated tailing [31–33]; the 5’ ppp, OH, and cap structure inhibit 5’ ligation [26,34]; the cyclic phosphate and 3’ phosphate at the 3’ end block 3’ ligation. For these modifications, special enzymatic treatments or cloning strategies are required for efficient cloning, usually leading to yield loss and requiring additional labor. Conversely, these modifications may provide markers for enzymatic selections and affinity-based purification, as detailed below.

In this review, we will compare different strategies for cloning modified and unmodified small RNA species and provide recommendations on best practice for constructing small RNA libraries.

## RNA QUALITY AND QUANTITY

Small RNA cloning can start with total RNAs or purified small RNAs. Many old protocols used purified small RNAs which can be obtained *via* denaturing polyacrylamide gel (PAGE) purification (6–8 M urea) or affinity-based purification [19,29,31]. The former can even reach single-nt resolution, generating small RNA fractions of better quality; the latter usually first utilizes affinity columns to remove big RNA and then column or alcohol precipitation to concentrate small RNAs of less than 200 nts [31]. In both cases, a denaturing condition containing urea or guanidine is preferred for separating small RNAs from their targets.

PAGE-based purification is still desirable for samples containing lots of degraded RNAs, which may overwhelm authentic small RNAs. For example, immunoprecipitated RNA samples may contain lots of degraded tRNAs, rRNAs, and mRNAs, and at least a small fraction of them are ligatable, likely generating a huge background noise. PAGE purification can remove this noise by selecting small RNAs of desired sizes. In addition, most cloning protocols have a limited amount of linkers and enzymes which are calculated based on intact RNAs. A sample containing lots of degraded RNAs may complicate the calculation and result in biased libraries due to limited linkers or enzymes and differential ligation efficiency of different small RNA species.

An affinity or alcohol precipitation based small RNA isolation process may substitute for PAGE-based purification. It is much more convenient and only requires a few chemical solutions. Many commercial and custom-made protocols are available [29,35,36]. In these protocols, small RNAs and big RNAs are usually differentially precipitated with different concentrations of alcohol or LiCl. Among them, the MirVana™ miRNA isolation kit is a pioneering commercial kit for enriching small RNAs of less than 200 nts long. As an affinity-based protocol, it requires two column purification steps: one for selecting big RNAs and the other for selecting small RNAs. The process is labor-intensive and the kit is very expensive. A simplified version by replacing column purification with alcohol precipitation was developed, basically reducing the cost and labor to negligible levels [29,31]. The MirVana kit lysis/binding buffer can be substituted with solution D [37]. The critical component of these solutions is the denaturing reagent, usually guanidine, for separating small RNAs from their targets, maximizing the yield of authentic small RNAs.

Since PAGE-purification is tedious and time-consuming, and may not be available in many labs, most latest commercial or custom-made protocols utilize total RNAs [38]. Obtaining total RNAs of high quality constitutes the first best practice since contamination of phenol, alcohol and denaturing reagents during RNA isolation processes often causes inhibitory effect on ligation and RT reactions. A OD260/280 ratio less than 1.8 may indicate protein contamination while a ratio more than 2.2 may indicate contamination of benzene-ring containing chemicals such as phenol.

A pure phenol (without chloroform)-based protocol can often result in phenol contamination since phenol itself has a significant solubility in aqueous solution. Phenol contamination can be observed visually over time when phenol is oxidized, generating a yellowish color. Therefore, it is always safe to reprecipitate samples and wash RNA pellets well with alcohol especially when a large volume of reagents is used. The alcohol washing step in many RNA isolation protocols may seem simple and straightforward. However, many researchers may not realize that their samples may be contaminated by alcohol especially when using low-binding tubes since surface tension in those tubes retains more fluid, though sometimes maybe invisible at a glance, on the inner surface. A good practice is to make a second spin after removing the bulk alcohol and then remove the residual completely.

## CLONING STRATEGIES

Many cloning strategies have been developed primarily due to special needs for cloning modified small RNAs or for simplifying cloning processes. Although RNAs can be directly sequenced without conversion to cDNA, the most popular sequencing platforms need cDNA libraries for RNA sequencing. In order to make such libraries, linkers are attached to the 5’ and 3’ ends of small RNAs so that these RNAs can be converted to cDNA and thereafter amplified using PCR.

**Figure 1:**
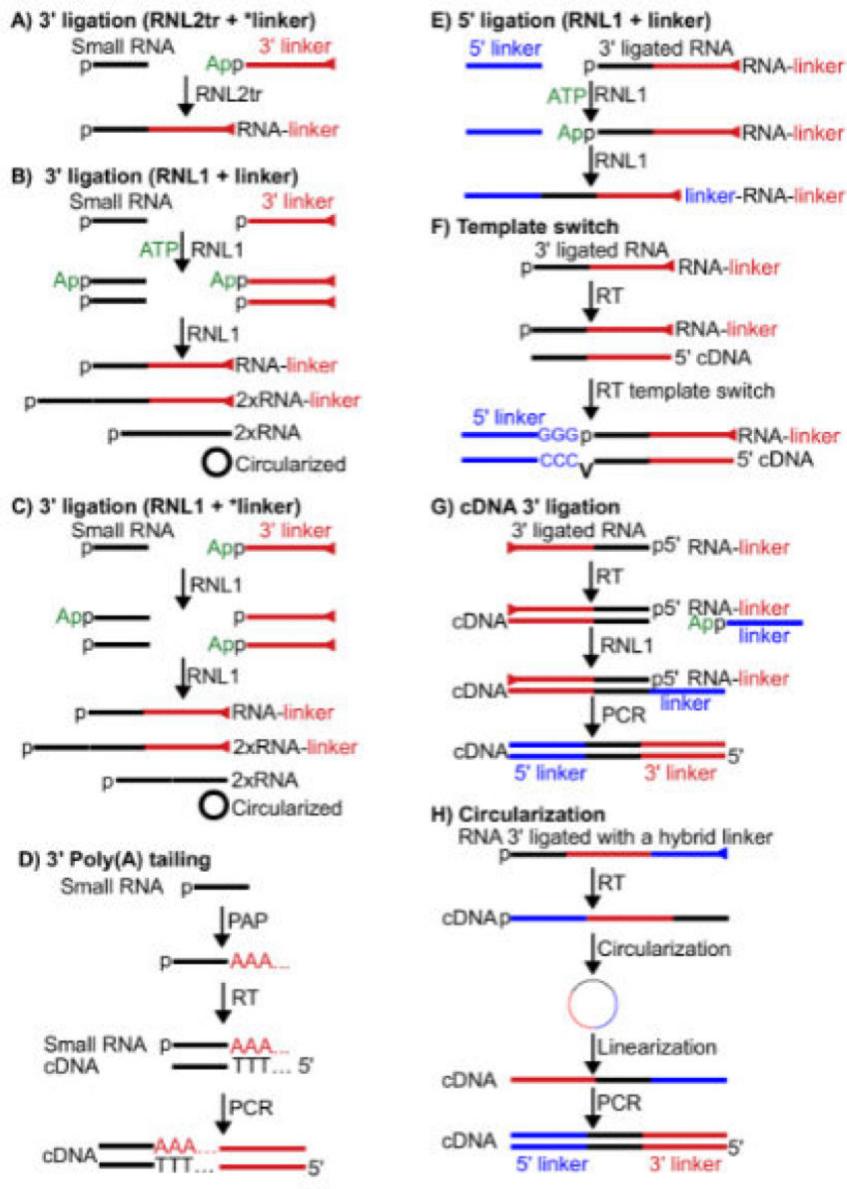
A-H Strategies to add 5’ and 3’ linkers to small RNAs. (A) Small RNAs are ligated by RNL2tr with an adenylylated 3’ linker; (B) Small RNAs are ligated by RNL1 with non-adenylylated 3’ linker and ATP, generating dimerized (2xRNA) and circularized small RNAs in addition to correct products; (C) Small RNAs are ligated by RNL1 with an adenylylated 3’ linker (no ATP), generating dimerized and circularized small RNAs in addition to correct products; (D) Small RNAs are poly(A)-tailed using PAP, converted to cDNA with a poly(T) oligo, extended (cDNA) by PCR for 3’ linker addition; (E) 5’ linkers are added to small RNAs using RNL1 with ATP; (**F)** 5’ linkers are added to small RNAs using RT template switch in which the untemplated CCC is added when RT reactions reach the 5’ end of template RNAs, annealed with the 3’ GGG of the 5’ linker, extended to the 5’ end of the 5’ linker; (G) an adenylylated linker complementary to the 5’ linker is added in a 3’ ligation reaction to the first strand cDNA reverse transcribed from the 3’ ligated RNA (represented as inverted), and then converted to the 5’ linker by PCR; (H) A hybrid 3’ and 5’ linker is attached to small RNAs, converted to the first strand cDNA, circularized and linearized to separate the 5’ and 3’ linker. In all figures, blue for 5’ linker, red for 3’ linker, and black for small RNAs. *linker, adenylylated; linker, non-adenylylated. RNL2tr, truncated T4 RNA ligase 2; RNL1, T4 RNA ligase 1; PAP, poly (A) polymerase. All nucleic acids are drawn from 5’ (left) to 3’ (right) unless otherwise indicated. Triangle at the 3’ end of linkers indicates a chemical modification blocking ligation.

Based on the chemistry, linkers can be added using ligation-dependent or independent manners [19,29–31,33]. The 3’ linker is usually added to small RNAs using ligation-dependent manner by RNA ligases. The truncated T4 RNA ligase 2 (RNL2tr) is preferred over the wild type (WT) T4 RNA ligase 1 (RNL1) [33]. Since RNL2tr lacks the adenylylation domain, it cannot activate the donor. Therefore, an activated (adenylylated) ssDNA oligo usually serves as the 3’ linker (donor; Figure 1A) and no ATP is required in the ligation reaction [39,40]. T4 RNA ligase 1 (RNL1) can substitute for RNL2tr [29]. However, it needs ATP and can activate (adenylylate) target small RNAs, converting them to donor RNAs and generating dimerized and circularized small RNA byproducts (Figure 1B). These byproducts can still be generated when using an adenylylated ssDNA oligo as 3’ linkers without ATP since RNL1 may transfer the adenylyl group or utilize contaminating ATP in RNL1 enzymes to activate target small RNAs, generating adenylylated small RNAs (Figure 1C) [29].

Not only do these byproducts reduce ligation yields and generate experimental artifacts, they also produce cloning bias since only small RNAs with a free 3’ OH group (not 2’-O-methylated, as discussed below) at the 3’ end can serve as acceptor molecules in these byproducts.

The 3’ linker can also be introduced using PAP followed by an RT-PCR reaction (Figure 1D) [41]. Since the Poly (A) tail size is not predictable, small RNA sequences ending with A’s may be truncated at the poly (A) tail removal step by the bioinformatical pipelines. Therefore, this tailing method cannot be used to analyzed small RNAs with 3’ untemplated nts, which regulate RNA stability in many organisms [29,42–45].

Adenylylated ssDNA oligos are commercially available but cost-prohibitive. For most labs, chemical synthesis may prove difficult mainly due to lack of appropriate equipment and safety concerns. Alternatively, these oligos can be synthesized using RNA or DNA ligases [38,46,47]. Since the synthesis is a single turnover reaction, a large amount of enzyme is needed. Fortunately the ligase clone is well spread in the science community and the purification process only costs a penny and requires minimum equipment and effort.

The 5’ linker is added to small RNAs using various strategies after 3’ ligation (Table 1). It can be directly ligated to 5’p-RNAs using RNL1 with the cofactor ATP (Figure 1E) [29,31,33,44]. For 5’ppp-RNAs, 5’OH-RNAs, and csRNAs, enzymatic pretreatment is needed to generate 5’p-RNAs for ligation, as discussed below.

The 5’ linker can be added to the first strand cDNA at the RT step. Since reverse transcriptases usually add a few non-templated nts after finishing template-based transcription, these nts (CCC in Figure 1F) can be utilized to anneal with a 5’ linker containing GGG at the 3’ end, leading to template switch for continuing cDNA synthesis based on the 5’ linker [29,44]. The template switch method does not require a 5’p structure and thus is able to clone RNAs with a 5’p, OH, ppp, and cap structure. This method is also capable of cloning truncated RT products caused by internal RNA modifications. However, this method may generate bias since the template switch step prefers small RNAs starting with G likely because the synthesis of non-templated CCC by RT is not very efficient and thus templated terminal C of cDNA (corresponding to 5’G of small RNAs) can be used to anneal with the 5’ linker [29].

The 5’ linker can be added after the first strand cDNA synthesis using another 3’ ligation with an adenylylated linker (Figure 1G) [30]. This adenylylated linker contains a sequence complementary to the 5’ linker and becomes 5’ ligated to small RNA sequences after PCR. Since this 5’ linker addition strategy bypasses the 5’p requirement, it can clone small RNAs with various 5’ ends. In addition, this method is capable of cloning truncated RT products caused by internal RNA modifications.

The 5’ linker can be added using a circularization method in which a hybrid (3’+5’) linker is 3’ ligated to small RNAs, reverse transcribed, circularized, and linearized (Figure 1H) [48,49]. Eventually, small RNA sequences are flanked with 5’ and 3’ linkers. This method also bypasses the 5’p requirement and is capable of cloning small RNAs with various 5’ end structures and truncated cDNA caused by internal RNA modifications. In summary, the RNA 5’ ligation method needs a 5’p on small RNAs, thereby selecting miRNAs, some siRNAs and piRNAs but excluding ppp-RNAs, csRNAs and 5’OH-RNAs. However, enzymatic treatment can be applied to convert non-5’-p-RNAs to 5’p-RNAs for 5’ ligation, as discussed below.

In contrast, the rest methods are capable of cloning small RNAs with various 5’ end structures. However, this versatility often causes confusion. For example, ppp-RNAs (nascent RNAs), csRNAs and 5’OH-RNAs may all be derived from the same 5’ end sequences of mRNAs. Cloning them as a pool may complicate any study focusing on a specific biological process. In addition, most functional small RNAs bear 5’p. To accurately quantify these RNAs, a 5’p selection, here as 5’ ligation, is desirable to enrich authentic RNAs while depleting other species, primarily degraded RNAs which usually contain 5’OH. Therefore, in most experiments, RNA 5’ ligation is likely the best practice.

## CLONING MODIFIED SMALL RNAs

**Figure 2:**
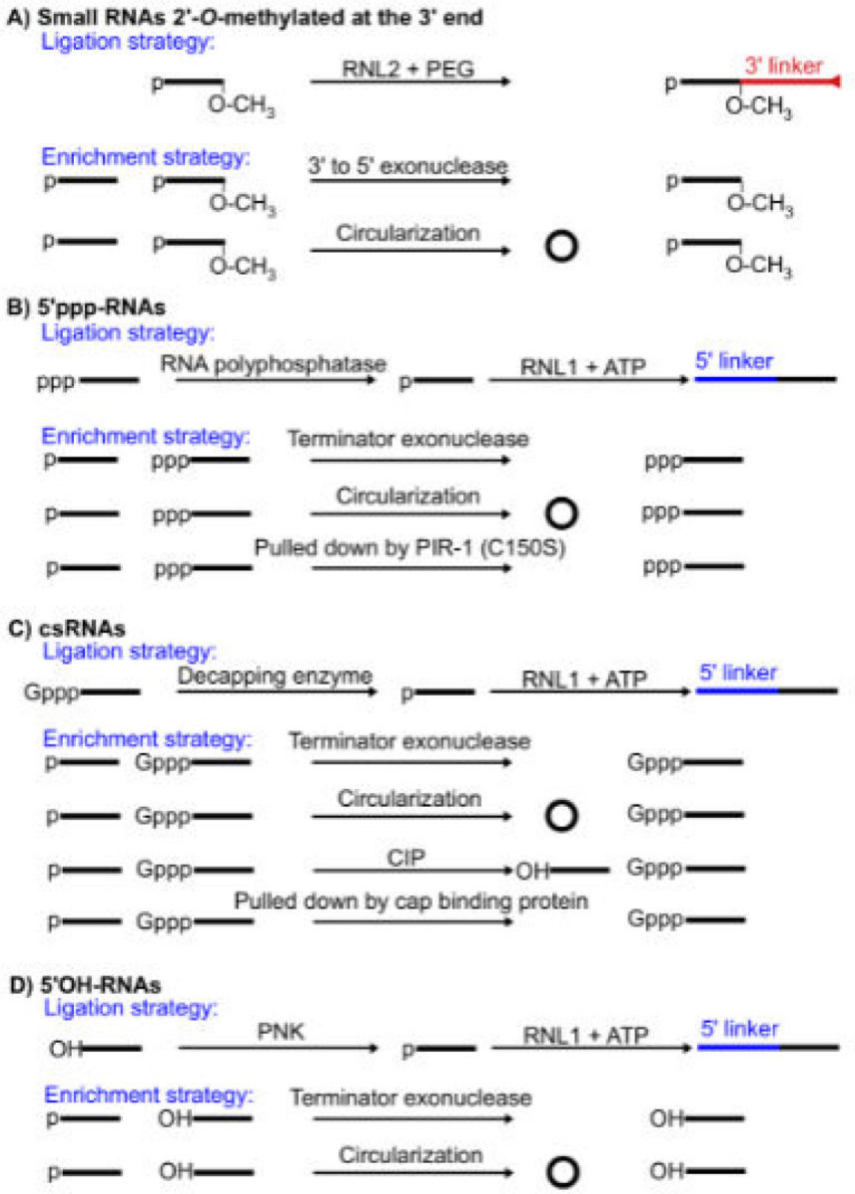
A-D Cloning modified small RNAs. (A) Strategies to enrich and ligate small RNAs 2’-Omethylated at the 3’ end. (B-D) Strategies to enrich 5’ modified small RNAs, as indicated, and to generate 5’p-RNAs from these RNAs for ligation. CIP, calf intestinal phosphatase; PNK, polynucleotide kinase; PEG, polyethylene glycol. Blue for 5’ linker and red for 3’ linker. Triangle at the 3’ end of linkers indicates a chemical modification blocking ligation.

To clone modified small RNAs, special strategies and treatment have been developed. Many small RNAs are 2’-O-methylated at the 3’ end for protection [19,27,50]. This modification inhibits ligation and PAP-mediated Poly (A) tailing. However, this inhibitory effect can be overcome using RNL2 with 25% Polyethylene glycol (PEG) and extended ligation time (Figure 2A) [29,33]. The RT step is still compromised even if PEG is added [33]. But the 70% read-through rate is acceptable. 2‘-Omethylation can be used for enriching RNAs since it is capable of protecting RNAs from 3’ to 5’ mediated degradation. Another enrichment method is to use circularization treatment to hide 3’OH, making 3’OH-RNAs incompatible for further 3’ ligation (Figure 2A).

5’ppp-RNAs can be dephosphorylated to 5’p-RNAs using polyphosphatases and then ligated (Figure 2B) [29]. C. elegans PIR-1 is an RNA polyphosphatase and works efficiently in ligation conditions while commercial polyphosphatases need special reaction conditions incompatible with ligation conditions [38]. This makes C. elegans PIR-1 a perfect choice in the one-pot cloning strategy for simultaneously cloning 5’p and ppp-RNAs in an all liquid based manner. 5’ppp-RNAs can be enriched by Terminator exonuclease which specifically destroys 5’p-RNAs, the bulk species of small RNAs, or by circularization, which makes 5’p-RNAs unligatable [29,31]. Since C. elegans PIR-1(C150C) mutant protein cannot dephosphorylate 5’ppp-RNAs and can bind 5’ppp-RNAs but not 5’p-RNAs tightly, it may be used as an affinity tool to purify 5’ppp-RNAs (Figure 2B) [38].

csRNAs can be decapped to 5’p RNAs using decapping enzymes and then ligated (Figure 2C) [23]. Since Tobacco Acid Pyrophosphatase was discontinued, several substitutes have been developed [38,51,52]. Among them, hDcp2 works efficiently in the ligation condition and was used in the one-pot cloning strategy for simultaneously cloning capped and non-capped RNAs [38].

csRNAs can be enriched by Terminator exonuclease or circularization using similar mechanisms as above; csRNAs can be pulled down using cap-binding proteins [53]; csRNAs can also be enriched by calf intestinal phosphatase (CIP), which dephosphorylates p-RNAs and ppp-RNAs, making them unligatable (Figure 2C) [23].

5’OH-RNAs can be phosphorylated using polynucleotide kinase (PNK) and then ligated (Figure 2D). Similarly 5’OH-RNAs can be enriched using terminator exonuclease and cicularization treatments.

Depending on RNA expression levels, many cloning strategies utilize a combination of appropriate ligation and enrichment strategies to maximize yields. 5’p-RNAs including miRNAs and piRNAs are usually expressed at relatively high levels and require no enrichment. So do 5’ppp-RNAs in C. elegans since they are the major small RNA species [29]. csRNAs are usually expressed at extremely low levels and can be enriched using Terminator exonuclease or CIP treatments before 5’ ligation (Table 2) [23]. The recommended strategies for cloning specific small RNA species are listed in Table 2. All these strategies may generate background noise primarily due to RNA secondary structures. For example, terminator exonuclease, CIP, and polyphosphatase may not completely remove or modify RNAs with a recessive 5’ end.

## PCR OVERCYCLING

**Figure 3:**
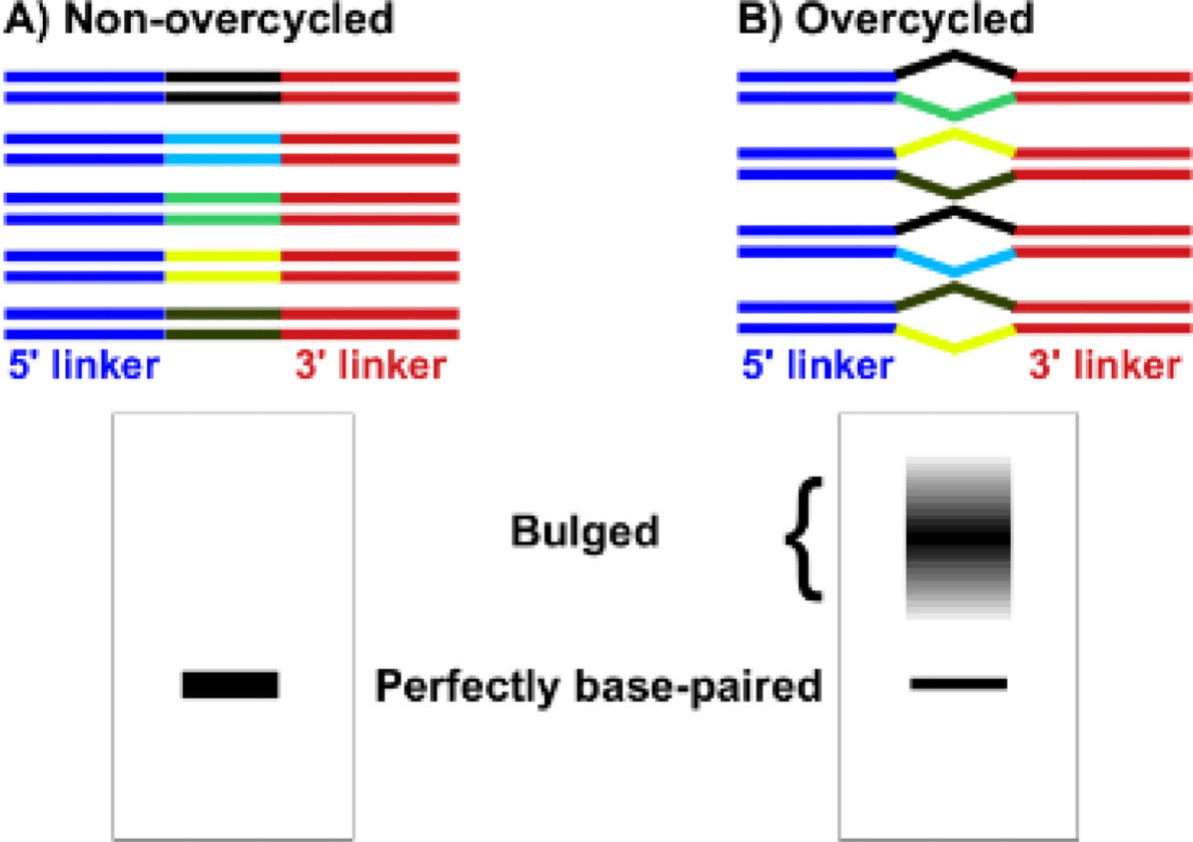
A-B PCR overcycling. Unlike non-overcycled PCR, which generates perfectly base-paired DNA visualized as a single band on the gel (A), overcycled PCR generates a perfectly base-paired DNA band with dramatically reduced intensity and a smear of bigger molecular weight containing bulged products (B). Blue for 5’ linker, red for 3’ linker, and all others for small RNAs.

PCR overcycling usually occurs when primers are used up, resulting in futile PCR cycles without further yield increase. Since most PCR reactions only generate one product, the denaturing and renaturing cycles after primers are used up only waste energy without generating undesirable byproducts. However, in small RNA libaries, cDNA amplicons share 5’ and 3’ linker sequences, which may constitute 85% of the sequences. When a PCR reaction runs out primers, these amplicons can anneal with each other using the shared end sequences, resulting in bulged products (Figure 3). This may dramatically reduce the band intensity at the expected size (Figure 3).

It is almost impossible to determine where to cut and if the cut area really represents the whole libraries. Moreover, if only the area at the expected size is cut, the libraries are significantly biased for abundant RNA species. For example, for a overcycled library consisting of 2 sequences, one at 99% and the other at 1%, 99% *99% of the major sequence and 1% *1% of the minor sequence are mapped at the expected size, constituting 99.99% and 0.01% respectively. It is obvious that the minor species is way underrepresented. What if just cutting the much bigger area consisting of both the expected size and the bulged product? First, it is hard to define the bulged product area since it appears like a smear. More importantly, the bulged products may contain a significant fraction of primer dimers and undesirable amplicons, such as those derived from tRNAs and rRNAs.

Commercial kits never examine if a PCR reaction is overcycled or not since most researchers will be satisfied if they can make a library. As for if a library is biased or not, it is hard to examine it anyway. A decent strategy was proposed recently to solve this issue without causing apparently more workload for library construction. The solution is to perform PCR reactions with 0.1 μM of primers for a certain number of cycles (16 in the protocol), pause the PCR to add 0.6 μM of the same primers, and continue the PCR reactions for one or two cycles [38]. This way, the PCR reaction, if overcycled, is restored by excessive newly added primers. If not, the PCR reaction won’t be overcycled with the additional primers anyway.

## PCR PRODUCT QUANTIFICATION

In all cloning protocols, target amplicons need to be gel-purified to remove primer dimers and byproducts such as tRNA and rRNA amplicons and then quantified. Barcoded samples are usually mixed as a pool for sequencing. This process is labor-intensive and time-consuming. It is highly desirable to quantify samples without purification, mixed and purified as a pool (one sample). The premise for this pooling method is to obtain the concentration of target amplicons in unpurified samples. One solution is to use Bioanalyzers. However, the resolution may not be enough to separate authentic small RNAs from degraded RNAs, such as tRNA and rRNA fragments. Moreover, this method is expensive and inconvenient since most labs cannot afford such equipment. A simple solution was provided recently. Barcoded samples can be compared side by side on a PAGE gel and quantified visually, mixed according to a desired ratio, concentrated, and purified as a mixed library ready for sequencing [38]. This way, ~20 samples can be purified as one sample, significantly reducing workload. The quantification may not be very accurate. However, the 2–3 fold variation is bearable for most experiments.

## ONE-POT METHODS

Commercial kits are usually only optimized to clone 5’pcontaining and 3’ unmodified small RNAs, such as some miRNAs and siRNAs. However, many biological samples contain modifications affecting 5’ and 3’ ligation. To efficiently and sometimes specifically clone these modified RNAs, enzymatic pretreatment is required. The cloning process may only take 2-hour labor for cloning 20 samples without pretreatment. However, pretreatment may easily double, triple, quadruple the labor time since most pretreatment steps involve incubation and RNA isolation. Moreover, these steps are labor-intensive. As reported recently, these pretreatment steps can be performed in ligation conditions. For example, dephosphorylation of ppp-RNAs can be performed in the 3’ ligation step using PIR-1; decapping can be performed using hDcp2 in the 5’ ligation step; PNK can be added in the 5’ ligation step to phosphorylate 5’OH-RNAs for ligation [38].

## HOME-MADE KITS

Small RNA cloning kits are really expensive and usually the cost of constructing small RNA libraries overruns that of sequencing. The benefits of using commercial kits include convenience (fully liquid-based) and reliability of supplies if funding is not a limiting factor. However, the kits may be only suitable for cloning unmodified RNAs. Since many details of the protocols are not shared, an unfortunate fact occurring more and more often, it is hard to make calculation or modification to adjust the condition. A recent article provided all the details for making home-made kits which provide convenience (one-pot and fully liquid-based), low (negligible) cost, and versatility for cloning unmodified and several modified small RNAs [38]. It only requires a few home-purified enzymes (RNL1, RNL2tr, and DNA polymerase PFU) all of which can be substituted by commercial enzymes (not recommended since many commercial enzymes exhibit lower quality than home-made ones). The sensitivity is much higher than that of commercial kits since it is capable of cloning small RNAs using 20 ng of total RNAs and has a potential of using even less.

## CONCLUSION

In summary, small RNA cloning is more complicated than mRNA cloning. This review focuses on some of the most popular techniques used for cloning modified and unmodified small RNAs and may help researchers decide what constitutes a practical and accurate method to best meet the need of analyzing small RNAs.

## Figures and Tables

**Table 1:** Methods for adding 5’ linkers to small RNAs with different 5’ end structures.

Methods	5’p-RNA	5’ppp-RNA	5’-OH RNA	5’ Gppp-RNA
RNA 5’ ligation	Yes	No	No	No
cDNA template switch	Yes	Yes	Yes	Yes
cDNA 3’ ligation	Yes	Yes	Yes	Yes
Circularization	Yes	Yes	Yes	Yes

**Table 2:** Recommended methods to clone modified small RNAs.

Target small RNAs	Ligation and enrichment strategies
5’p-RNA only	5’ ligation
5’p+5’ppp-RNA only	Polyphosphatase → 5’ ligation
5’ppp-RNA only	Terminator → polyphosphatase → 5’ ligation
csRNAs only	Terminator or CIP → decapping enzyme → 5’ ligation
csRNAs+5’p-RNAs only	decapping enzyme → 5’ ligation
5’OH-RNA only	Terminator → PNK → 5’ ligation
All	5’ ligation-independent cloning

1. Terminator exonuclease destroys 5’p-RNAs; 2. Polyphosphatase generates 5’p-RNAs from 5’pp or ppp-RNAs; 3. Decapping enzyme generates 5’p-RNAs from csRNAs.
